# The potential effects of HECTD4 variants on fasting glucose and triglyceride levels in relation to prevalence of type 2 diabetes based on alcohol intake

**DOI:** 10.1007/s00204-022-03325-y

**Published:** 2022-06-17

**Authors:** Yoo Jeong Lee, Hansongyi Lee, Han Byul Jang, Min-Gyu Yoo, Sumin Im, Soo Kyung Koo, Hye-Ja Lee

**Affiliations:** Division of Endocrine and Kidney Disease Research, Department of Chronic Disease Convergence Research, National Institute of Health, Cheongju, 28159 Chungbuk Korea

**Keywords:** HECTD4, SNPs, Type 2 diabetes, Alcohol consumption

## Abstract

Excessive alcohol intake is an important cause of major public health problem in East Asian countries. Growing evidence suggests that genetic factors are associated with alcohol consumption and the risk for alcohol-associated disease, and these factors contribute to the risk of developing chronic diseases, including diabetes. This study aims to investigate the association of type 2 diabetes with genetic polymorphisms within *HECTD4* based on alcohol exposure. We performed a genome-wide association study involving the cohorts of the KoGES-HEXA study (*n* = 50,028) and Ansan and Ansung study (*n* = 7,980), both of which are prospective cohort studies in Korea. The top three single-nucleotide polymorphisms (SNPs) of the *HECTD4* gene, specifically rs77768175, rs2074356 and rs11066280, were found to be significantly associated with alcohol consumption. We found that individuals carrying the variant allele in these SNPs had lower fasting blood glucose, triglyceride, and GGT levels than those with the wild-type allele. Multiple logistic regression showed that statistically significant associations of *HECTD4* gene polymorphisms with an increased risk of type 2 diabetes were found in drinkers. Namely, these SNPs were associated with decreased odds of diabetes in the presence of alcohol consumption. As a result of examining the effect of alcohol on the expression of the *HECTD4* gene, ethanol increased the expression of HECTD4 in cells, but the level was decreased by NAC treatment. Similar results were obtained from liver samples of mice treated with alcohol. Moreover, a loss of HECTD4 resulted in reduced levels of CYP2E1 and lipogenic gene expression in ethanol-treated cells, while the level of ALDH2 expression increased, indicating a reduction in ethanol-induced hepatotoxicity.

**Supplementary Information:**

The online version contains supplementary material available at 10.1007/s00204-022-03325-y.

## Introduction

Diabetes is a major health issue that has an alarming prevalence worldwide. In 2019, it was estimated that 463 million people had diabetes, and this number is projected to reach 578 million by 2030 and 700 million by 2045 (Saeedi et al. 2019). Type 2 diabetes is associated with an increased risk of cardiovascular disease, such as myocardial infarction, stroke, and clinical complications, leading to mortality.

Several epidemiological studies have suggested that alcohol is a risk factor for type 2 diabetes, supporting evidence that alcohol consumption is an environmental factor influencing glycemic control. Moreover, many studies have shown that modifications of lifestyle risk factors, including obesity, physical inactivity, and excessive alcohol use, are effective in preventing type 2 diabetes in high-risk adults with impaired glucose tolerance (Zimmet et al. 2001). Although moderate alcohol consumption is associated with a reduced risk of type 2 diabetes, several studies have shown that any amount of alcohol consumption is not beneficial and can increase the risk of diabetes (Baik and Park 2020; Flanagan et al. 2002). It is likely that the divergent findings of previous studies are due to differences in alcohol drinking patterns, alcohol use frequency, and genetic factors interacting with different environments.

The accumulated findings indicate that alcohol use phenotypes, such as initiation of use, maximum number of drinks consumed in a day, and initial response to alcohol, are genetically influenced (Agrawal et al. 2011; Fowler et al. 2007). In addition, the genetic architecture underlying many different alcohol-related phenotypes has been used to investigate diagnostic alcohol outcomes. However, replicated evidence of loci has been limited mostly to alcohol metabolizing genes; for instance, rs1229984 in *ADH1B* (alcohol dehydrogenase 1B) and rs671 in *ALDH2* (aldehyde dehydrogenase 2) are protective against greater alcohol consumption (Kimura and Higuchi 2011). Recently, a meta-analysis of significant genome-wide association studies on alcohol consumption identified that *ADH1B* (rs1229984), *KLB* (rs13130794), *BTF3P13* (rs144198753), *GCKR* (rs1260326), *SLC39A8* (rs13107325), and *DRD2* (rs11214609) are significantly associated with alcohol consumption that the risk of disease increases with increased alcohol consumption in white British individuals (Thompson et al. 2020). In particular, *β-Klotho* polymorphism rs11940694 was strongly associated with alcohol consumption in populations of European descent (Schumann et al. 2016). However, few such genetic studies have examined the effect of alcohol consumption, especially in Koreans. Therefore, we conducted a genome-wide association study (GWAS) on the effect of alcohol consumption in the Korean population.

In the present study, we identified genetic variants in *HECTD4* (HECT domain e3 ubiquitin protein ligase 4) that were associated with alcohol consumption in large population-based study. *HECTD4* rs77768175, rs2074356 and rs11066280 polymorphisms were associated with decreased fasting blood glucose and triglyceride (TG) levels. Moreover, we investigated the association between *HECTD4* polymorphisms and the risk of type 2 diabetes according to drinking status, and statistically significant associations of *HECTD4* gene-polymorphisms with increased risk of type 2 diabetes were found in drinkers. To identify the impact of drinking on *HECTD4* function, we then carried out gene expression and cellular functional studies in SK-Hep1 cells. Here, we showed that hepatic expression of HECTD4 was induced upon ethanol treatment, and the deletion of the *HECTD4* gene ameliorated ethanol-induced cell damage.

## Materials and methods

### Study design and participants

The Korean Associated Resource project (KARE) was initiated to examine the correlation between environmental and genetic factors and common chronic diseases (Cho et al. 2009). In this study, data from two population studies, the HEXA study and the Ansan–Ansung cohort study, conducted by the Korean National Institute of Health as part of the Korean Genome Epidemiology study (KoGES), were made accessible to the KARE. Detailed information can be found elsewhere (Kim and Han 2017).

The HEXA study recruited individuals over 40 years old living in urban cities (Seoul, Busan, Daegu, Gwangju, Ulsan, Anyang, Gyeonggi province, Chuncheon, Gangwon province, Cheonan, Chungnam province, Masan, and Gyeongnam province) from 2004 to 2013 and followed up with these individuals. The Ansan–Ansung cohort study recruited individuals aged 40–69 years living in Ansan (urban area) and Ansung (rural area) from 2001 to 2002. After the quality control of genotype, we excluded subjects having missing genotype from the HEXA (*n* = 58,700) and Ansan–Ansung (*n* = 8,840) cohorts. We also eliminated subjects who had a diagnosis of any cancer (HEXA = 1318; Ansan–Ansung = $6$), missing information on alcohol consumption (HEXA = 2,695; Ansan–Ansung = 827), or missing information on diabetes diagnosis (HEXA = 1261; Ansan–Ansung = 27). Finally, 50,028 individuals from the HEXA cohort and 7,980 from the Ansan–Ansung cohort study were included in the final analysis (Fig. S1). The study protocol was approved by the Institutional Review Board of the Korea Disease Control and Prevention Agency (KDCA) (2019–03-01-PE-A).

### Screen for genetic variants

DNA samples from the HEXA and Ansan–Ansung studies were genotyped using Affymetrix Genome-Wide Human SNP Arrays 6.0 and 5.0, respectively (Affymetrix Inc., Santa Clara, CA, USA) and processed using a Bayesian robust linear model with the Mahalanobis distance genotyping algorithm. In the 24,080 (HEXA) and 6,522 (Ansan–Ansung) SNPs identified, the relationship between *HECTD4* and alcohol consumption was tested with a generalized logistic regression additive model and a Bonferroni correction. Following Bonferroni correction, 143 (2.1 × 10–6) and 2547 (7.7 × 10–6) SNPs were significantly associated with alcohol consumption in the HEXA and Ansan–Ansung groups, respectively. Finally, we selected three SNPs (rs77768175, rs2074356, and rs11066280) in *HECTD4* as candidate genetic variants.

According to *P* values, we identified the top 20 SNPs (Supplementary Table 1) showing more significant association with alcohol consumption in this study. We confirmed *ALDH2, HECTD4, MYL2, CCDC63, OAS3,* and *RPH3A* in the HEXA dataset and *ALDH2* and *HECTD4* in the Ansan–Ansung dataset. Of these, *ALDH2* polymorphisms are already well known to be alcohol metabolism enzymes and affect alcohol consumption, but *HECTD4* was assessed in a small sample of male drinkers in Korea (*n* = 2834) (Baik et al. 2011); therefore, we need to confirm these findings in a larger population.

### Assessment of alcohol consumption

Data on alcohol consumption at baseline were collected using interview-based questionnaires. Participants were asked whether they consumed at least one alcoholic drink every month; if they had, they were asked whether they were former drinkers or current drinkers. In the case of current -drinkers, they were additionally asked to complete a questionnaire that inquired about the amount and frequency of alcohol consumed in the past 30 days. A total daily alcohol consumption was calculated using the average frequency, amount per occasion, and alcohol content of one standard drink. Participants who did not consume alcohol at baseline were classified as nondrinkers, and those who consumed alcohol were classified as drinkers. Additionally, we classified the participants by the amount of alcohol consumption into four groups as: non-drinkers, low (< 5 g/day), moderate (5–30 g/day), and high (≥ 30 g/day) drinkers.

### Definition of diabetes

Diabetes was defined as a fasting blood glucose level ≥ 126 mg/dl. Control was defined as a blood glucose level < 100 mg/dl among those without prediabetes or diabetes.

### Anthropometric and biochemical measurements

The general characteristics (age, sex, drinking status, and smoking status) and anthropometrics (height, weight, body mass index, waist circumference, and hip circumference) of the participants were examined by professionally trained personnel using standardized protocols. Body mass index (BMI) and waist–hip ratio (WHR) were calculated by the following formula: BMI = weight (kg)/height (m^2^), (WHR) = waist/hip. Data on aspartate aminotransferase (AST), alanine aminotransferase (ALT), alkaline phosphatase, gamma-glutamyl transferase (GGT), systolic and diastolic blood pressure, and blood parameters related to glucose were obtained from the KoGES database. The levels of total cholesterol, triglycerides, and high-density lipoprotein (HDL)-cholesterol were measured using an Advia 1650 analyzer (Siemens, Tarrytown, NY, USA).

### Animal experiment

For analysis of HECTD4 gene expression in alcohol-fed mice, we used liver tissue from a previously published study (Kim et al. 2014). Male C57BL/6 mice (6-week-old) were obtained from the Jackson Laboratory (Bar Harbor, ME), and subjected to 5% (v/v) ethanol administration for 6 weeks. All animals were cared for in accordance with the Korea National Institute of Health guidelines and approved by Korea National Institute of Health Animal Care and Use Committee.

### Quantitative PCR analysis

Total RNA was extracted from SK-Hep1 cells using an RNeasy Mini kit (Qiagen) according to the manufacturer's instructions. RNA concentrations were determined via a NanoDrop® (Thermo Fisher Scientific, Waltham, MA, USA). cDNA was synthesized using SuperScript III reverse transcriptase (Invitrogen; Thermo Fisher Scientific, Inc.). Quantitative PCR was performed using SYBR Green Master Mix (Thermo Fisher Scientific) in a total volume of 20 µL with the QuantStudio 6 Flex system (Thermo Fisher Scientific). The expression of the target genes was normalized to β-actin expression, and relative quantification was evaluated with the 2 − ΔΔCt method.

### Western blot analysis

Whole-cell lysates were extracted in RIPA extraction buffer, and protein concentrations were determined using a BCA protein assay (Pierce; Thermo Fisher Scientific, Inc., Waltham, MA, USA). Cell lysates (30 μg) were mixed with 20 μL of SDS-PAGE sample buffer and loaded on 4–12% SDS-PAGE gels. Antibodies against HECTD4 (MBP1-31,944) were obtained from Novus Biological (Centennial, CO, USA). Antibodies against β-actin (#8457), phospho-JNK (#4668), phospho-Ikb (#9246), and phospho-p65 (#3033) were purchased from Cell Signaling Technology (Beverly, MA, USA).

### Statistical analysis

To select SNPs associated with alcohol consumption, PLINK (ver. 1.9, Inc., Boston, USA) and SAS (ver. 9.4, Institute, Inc., Cary, NC) software were used. The association of imputed SNPs with alcohol consumption was analyzed with a multiple linear regression model after adjusting for age, sex and BMI. Data for categorical variables (sex, smoking, drinking status and diabetes status) of the subjects are expressed as frequencies and percentages, and a Chi-squared test was carried out for comparisons between groups. Other continuous variables (age, BMI) were calculated as the mean and standard deviation, and t tests were conducted. Additionally, the differences in fasting blood glucose levels according to SNPs were assessed using a general linear model after adjusting for covariates (age, smoking status, and BMI). To determine the association between HECTD4 carrying major homozygous genotypes vs. heterozygous genotypes and minor homozygous genotypes by alcohol consumption in relation to the risk of diabetes prevalence, multivariable logistic analysis was used, after adjusting for age, BMI, and smoking status. Values are presented as odds ratios (ORs) with 95% confidence intervals (CIs). Statistical significance was indicated by *p* < 0.05.

Statistical analyses were performed using GraphPad Prism software (GraphPad, San Diego, CA, USA). Comparisons between the two groups were performed using Student's *t* test or the nonparametric Mann–Whitney *U* test. For multiple-group comparisons, one-way analysis of variance (ANOVA) with Tukey's post hoc test for multiple comparisons was used to evaluate significant differences. Values of *p* < 0.05 were considered statistically significant.

## Results

### Baseline characteristics of the study subjects according to alcohol consumption status

Baseline data were obtained from two population-based cohorts as follows: 50,028 adults in the KoGES-HEXA study and 7980 adults in the KoGES-Ansan and Ansung study. The general characteristics of the study population are shown in Table 1. The participants were divided based on drinking status as follows: nondrinkers, 27,149 (54.3%) and drinkers, 22,879 (45.7%) in the HEXA group; nondrinkers, 4044 (50.7%) and drinkers, 3936 (49.3%) in the Ansan–Ansung group. The mean age of the subjects was 53.6 ± 8.0 years in the HEXA group and 52.0 ± 8.9 years in the Ansan–Ansung group. The proportion of drinkers was higher in men than in women. The vast majority of nondrinkers were nonsmokers, whereas smoking was observed more frequently in drinkers than in nondrinkers. Drinkers had higher fasting blood glucose levels, higher systolic and diastolic blood pressures, and higher HDL than nondrinkers on average. In particular, TG levels were considerably higher in drinkers than in nondrinkers. Moreover, the mean levels of serum AST and GGT were significantly higher in drinkers than in nondrinkers. However, there were no significant differences in the prevalence of diabetes between drinkers and nondrinkers.

**Table 1:** Characteristics of the study populations

	HEXA (*n* = 50,028)		*P*-value	Ansan–Ansung (*n* = 7980)		*P*-value
	Non-drinker	Drinker		Non-drinker	Drinker	
Age (years)	54.5 ± 7.8	52.6 ± 8.0	< .0001	53.5 ± 9.0	50.5 ± 8.5	< .0001
Number of subjects (*n*,%)	27,149 (54.3)	22,879 (45.7)	< .0001	4044 (50.7)	3936 (49.3)	
Male (*n*,%)	3775 (13.9)	12,832 (56.1)	< .0001	764 (18.9)	2850 (72.4)	< .0001
Female (*n*,%)	23,374 (86.1)	10,047 (43.9)		2850 (81.1)	1086 (27.6)	
Smoking status (*n*,%)			< .0001			< .0001
Non-smoker	24,682 (91.0)	12,522 (54.8)		3415 (85.3)	1425 (36.3)	
Former smoker	1430 (5.3)	5835 (25.5)		245 (6.1)	879 (22.4)	
Current smoker	1022 (3.8)	4507 (19.7)		346 (8.6)	1615 (41.2)	
Diabetes (%)	2293 (8.45)	1989 (8.7)	0.3241	370 (9.2)	349 (8.9)	0.6595
Body mass index (kg/m^2^)	23.7 ± 2.9	24.0 ± 2.7	< .0001	24.7 ± 3.2	24.5 ± 3.0	0.0002
GGT (IU/L)	23.2 ± 21.8	38.4 ± 49.1	< .0001	22.7 ± 22.6	53.8 ± 89.3	< .0001
AST (IU/L)	23.4 ± 29.8	24.1 ± 12.1	0.0001	23.9 ± 18.3	28.5 ± 19.6	< .0001
ALT (IU/L)	21.5 ± 25.7	23.3 ± 18.5	< .0001	21.9 ± 29.7	27.3 ± 23.0	< .0001
Fasting blood glucose (mg/dl)	93.7 ± 18.5	96.3 ± 20.2	< .0001	91.3 ± 22.0	94.2 ± 24.1	< .0001
Systolic blood pressure (mmHg)	121.6 ± 14.9	123.4 ± 14.6	< .0001	121.8 ± 19.2	121.3 ± 17.9	0.2315
Diastolic blood pressure (mmHg)	74.8 ± 9.5	76.8 ± 9.8	< .0001	79.3 ± 11.4	81.1 ± 11.4	< .0001
Triglycerides (mg/dl)	118.6 ± 74.9	133.3 ± 97.1	< .0001	143.2 ± 96.2	164.4 ± 124.5	< .0001
Total cholesterol (mg/dl)	198.9 ± 35.9	196.6 ± 35.1	< .0001	199.3 ± 36.5	198.9 ± 37.2	0.6562
HDL-cholesterol (mg/dl)	53.8 ± 12.8	54.1 ± 13.5	0.0174	44.1 ± 9.8	45.7 ± 10.3	< .0001
HECTD4 genotype (%)						
rs77768175 (AA/AG/GG)	58.9/37.1/4.0	83.6/16.1/0.3	< .0001	546/40.0/5.4	81.4/18.0/0.6	< .0001
rs2074356 (GG/GA/AA)	57.2/38.3/4.5	81.3/18.2/0.6	< .0001	58.9/36.7/4.4	85.0/14.8/0.2	< .0001
rs11066280 (TT/TA/AA)	62.1/34.6/3.4	85.1/14.7/0.2	< .0001	54.6/40.1/5.4	81.5/18.0/0.6	< .0001

Data are expressed as means ± standard deviations. Student’s *t* tests for continuous variables and Chi-square test for categorical variables were used to determine differences between groups. *HEXA* Health Examinees Study, *AST* aspartate aminotransferase, *ALT* alanine aminotransferase, *GGT* gamma-glutamyl transferase

### SNPs associated with alcohol consumption

We then performed an analysis of the association of single-nucleotide polymorphisms (SNPs) for alcohol consumption (g/day) using the Bonferroni correction in this Korean population. The top 20 SNPs that were associated with alcohol consumption are summarized (Table S1). Of these, the rs77768175, rs2074356 and rs11066280 SNPs within *HECTD4* showed strong evidence of an association with alcohol consumption. The data for the genotype and allele frequencies of three SNPs are shown in Tables 1 and 2, respectively. We examined the genetic effect of polymorphisms on alcohol consumption using linear regression analysis after adjusting for age, sex, and body mass index (BMI). As shown in Table 2, all three *HECTD4* variants were strongly correlated with decreased alcohol consumption in the study population.

**Table 2:** The association of HECTD4 gene with alcohol consumption

SNP	CHR	Position	Minor alleles	HEXA			Ansan–Ansung		
				MAF	β ± S.E	*P* value	MAF	β ± S.E	*P* value
rs77768175	12	112,736,118	G	0.16	– 0.84 ± 0.02	< .0001	0.17	– 0.80 ± 0.05	< .0001
rs2074356	12	112,645,401	A	0.14	– 0.83 ± 0.02	< .0001	0.15	– 0.95 ± 0.05	< .0001
rs11066280	12	112,817,783	A	0.17	– 0.73 ± 0.02	< .0001	0.17	– 0.81 ± 0.05	< .0001

*SNP* single-nucleotide polymorphism, *Chr* chromosome, *MAF* minor allele frequency, *HEXA* Health Examinees Study, β ± S.E, the effect size on the alcohol intake (natural log-transformed) in linear regression model after adjust age, sex, smoking status, and BMI

### Effect of HECTD4 variants on metabolic parameters according to drinking status

Next, we investigated whether these genetic variants in *HECTD4* were associated with metabolic parameters and classified them into three groups by the genotypes rs77768175, rs2074356 and rs11066280 based on alcohol drinking status. Among drinkers, the rs77768175 G-allele, rs2074356 A-allele, or rs11066280 A-allele carriers at *HECTD4* had significantly lower fasting blood glucose levels in both populations (Table 3). Moreover, similar results were observed for several metabolic traits related to type 2 diabetes, including WHR, triglyceride concentrations, HDL, and systolic blood pressure (SBP). Notably, alcohol drinkers showed higher levels of plasma TG than nondrinkers in the major allele homozygous genotype for rs77768175, rs2074356, and rs11066280. Meanwhile, among subjects who were homozygous for the minor alleles rs77768175 and rs11066280, drinkers showed lower levels of plasma TG than nondrinkers in the KoGES-Ansan and Ansung cohort. When evaluating the levels of ALT, AST, and GGT by alcohol consumption status, we observed that lower serum liver enzyme concentrations were associated with the rs77768175 G-allele, rs2074356 A-allele, and rs11066280 A-allele (Table 4 and Supplementary Table 2).

**Table 3:** Metabolic parameters according to HECTD4 genotype and alcohol consumption status

	HEXA				Ansan–Ansung			
*Fasting blood glucose (mg/dl)*								
*rs77768175*	AA	AG	GG	*P*-value	AA	AG	GG	*P*-value
Non-drinker	93.6 ± 18.1	93.8 ± 19.3	93.5 ± 15.5	0.2538	91.3 ± 21.4	91.3 ± 22.6	91.4 ± 24.0	0.9866
Drinker	96.8 ± 20.7	94.0 ± 16.9	92.4 ± 16.2	< .0001	94.9 ± 25.3	91.0 ± 17.9^b^	93.7 ± 23.6	0.0003
*rs2074356*	GG	GA	AA	*P*-value	GG	GA	AA	*P*-value
Non-drinker	93.7 ± 18.4	93.7 ± 18.8	93.7 ± 15.5	0.1961	91.3 ± 21.2	91.5 ± 23.1	90.5 ± 24.0	0.7456
Drinker	96.8 ± 20.7	93.8 ± 16.8^b^	93.7 ± 18.4	< .0001	94.8 ± 25.0	90.6 ± 18.5	87.4 ± 8.5	0.0003
*rs11066280*	TT	TA	AA	*P*-value	TT	TA	AA	*P*-value
Non-drinker	93.6 ± 18.1	93.9 ± 19.3	93.3 ± 15.3	0.1116	91.3 ± 21.4	91.3 ± 22.6	91.5 ± 24.1	0.9641
Drinker	96.8 ± 20.8	94.2 ± 17.1^b^	92.4 ± 15.0	< .0001	94.9 ± 25.3	91.0 ± 17.9	93.7 ± 24.1	0.0003
*Waist to hip ratio*								
*rs77768175*	AA	AG	GG	*P*-value	AA	AG	GG	*P*-value
Non-drinker	0.85 ± 0.06	0.85 ± 0.06	0.86 ± 0.06	0.0588	0.88 ± 0.08	0.88 ± 0.08	0.88 ± 0.07	0.3614
Drinker	0.87 ± 0.07	0.87 ± 0.06	0.86 ± 0.06	< .0001	0.88 ± 0.07	0.88 ± 0.07	0.87 ± 0.06	0.0112
*rs2074356*	GG	GA	AA	*P*-value	GG	GA	AA	*P*-value
Non-drinker	0.85 ± 0.06	0.85 ± 0.06	0.85 ± 0.06	0.0394	0.88 ± 0.08	0.88 ± 0.08	0.87 ± 0.07	0.1409
Drinker	0.87 ± 0.07	0.87 ± 0.06	0.86 ± 0.06	< .0001	0.88 ± 0.07	0.87 ± 0.07	0.85 ± 0.06	0.0015
*rs11066280*	TT	TA	AA	*P*-value	TT	TA	AA	*P*-value
Non-drinker	0.85 ± 0.06	0.85 ± 0.06	0.86 ± 0.06	0.0337	0.88 ± 0.08	0.88 ± 0.08	0.87 ± 0.07	0.1364
Drinker	0.87 ± 0.07	0.87 ± 0.07	0.86 ± 0.06	< .0001	0.88 ± 0.07	0.88 ± 0.07	0.87 ± 0.06	0.0106
*Triglycerides (mg/dl)*								
*rs77768175*	AA	AG	GG	*P*-value	AA	AG	GG	*P*-value
Non-drinker	116.7 ± 72.9	121.0 ± 77.4	124.0 ± 78.2	0.3654	142.3 ± 96.7	145.4 ± 97.9	136.6 ± 77.1	0.2293
Drinker	134.4 ± 99.9	126.3 ± 81.3	121.7 ± 55.8	< .0001	167.5 ± 126.5	151.4 ± 115.7	128.4 ± 72.5	0.0004
*rs2074356*	GG	GA	AA	*P*-value	GG	GA	AA	P
Non-drinker	116.9 ± 73.0	121.0 ± 77.6	125.5 ± 79.6	0.2839	141.5 ± 93.8	147.5 ± 102.4	131.2 ± 70.2	0.0253
Drinker	134.3 ± 99.7	125.9 ± 80.9	122.1 ± 58.5	< .0001	167.2 ± 127.4	148.4 ± 105.6	132.4 ± 95.4	0.0002
*rs11066280*	TT	TA	AA	*P*-value	TT	TA	AA	P
Non-drinker	116.6 ± 72.9	121.1 ± 77.3	122.8 ± 78.3	0.6664	142.4 ± 96.7	145.2 ± 97.9	137.2 ± 77.4	0.2877
Drinker	134.5 ± 99.9	126.9 ± 83.9	122.4 ± 60.2	< .0001	167.5 ± 126.5	151.6 ± 115.8	123.7 ± 70.6	0.0004
*High-density lipoprotein-cholesterol (mg/dl)*								
*rs77768175*	AA	AG	GG	*P*-value	AA	AG	GG	*P*-value
Non-drinker	54.4 ± 12.8	53.0 ± 12.7	51.8 ± 12.3	0.2538	44.4 ± 9.8	43.7 ± 9.8	43.5 ± 9.1	0.2621
Drinker	54.5 ± 13.6	51.9 ± 12.7	50.5 ± 11.3	< .0001	46.1 ± 10.6	43.9 ± 9.2	44.7 ± 6.9	< .0001
*rs2074356*	GG	GA	AA	*P*-value	GG	GA	AA	*P*-value
Non-drinker	54.3 ± 12.8	53.0 ± 12.7	51.6 ± 12.3	0.1961	44.3 ± 9.8	43.6 ± 9.7	44.4 ± 9.5	0.0495
Drinker	54.4 ± 13.6	51.9 ± 12.7	49.2 ± 10.2	< .0001	46.1 ± 10.6	43.4 ± 8.6	47.3 ± 7.6	< .0001
*rs11066280*	TT	TA	AA	*P*-value	TT	TA	AA	*P*-value
Non-drinker	54.4 ± 12.8	53.1 ± 12.7	52.0 ± 12.4	0.1116	44.4 ± 9.8	43.7 ± 9.8	43.5 ± 9.2	0.2857
Drinker	54.5 ± 13.6	52.4 ± 12.9	51.4 ± 12.1	< .0001	46.1 ± 10.6	43.8 ± 9.1	45.0 ± 6.8	< .0001
*Systolic blood pressure (mmHg)*								
*rs77768175*	AA	AG	GG	*P*-value	AA	AG	GG	*P*-value
Non-drinker	121.8 ± 15.1	121.3 ± 14.6	122.2 ± 14.6	0.1422	122.3 ± 19.7	121.1 ± 18.6	121.6 ± 18.7	0.5885
Drinker	123.6 ± 14.7	122.3 ± 14.0	121.1 ± 11.7	< .0001	121.8 ± 18.2	119.3 ± 16.5	112.4 ± 15.0	0.0030
*rs2074356*	GG	GA	AA	*P*-value	GG	GA	AA	*P*-value
Non-drinker	121.7 ± 15.1	121.3 ± 14.6	122.6 ± 14.7	0.0673	122.2 ± 19.7	121.2 ± 18.4	121.1 ± 19.1	0.8594
Drinker	123.5 ± 14.7	122.4 ± 14.0	122.3 ± 11.8	< .0001	121.9 ± 18.2	118.1 ± 16.1	116.3 ± 16.4	0.0003
*rs11066280*	TT	TA	AA	*P*-value	TT	TA	AA	*P*-value
Non-drinker	121.7 ± 15.1	121.4 ± 14.6	122.0 ± 14.6	0.4552	122.3 ± 19.7	121.1 ± 18.6	121.5 ± 18.6	0.6203
Drinker	123.6 ± 14.7	122.5 ± 14.1	122.2 ± 12.7	< .0001	121.8 ± 18.2	119.1 ± 16.4	112.8 ± 15.2	0.0020

Data are expressed as means ± standard deviations. Differences among the genotype groups were assessed by general linear models with adjustment for age, smoking status (non, former, current), BMI and fasting blood glucose. *HEXA* Health Examinees Study

**Table 4:** Blood liver parameters according to HECTD4 genotype and alcohol consumption status

	HEXA				Ansan–Ansung			
*Gamma-glutamyl transferase (GGT, IU/L)*								
*rs77768175*	AA	AG	GG	*P*-value	AA	AG	GG	*P*-value
Non-drinker	23.1 ± 22.1	23.2 ± 21.4	23.5 ± 20.8	< .0001	21.9 ± 21.1	23.5 ± 25.2	23.6 ± 17.3	0.2132
Drinker	40.0 ± 51.7	30.6 ± 32.0	25.6 ± 14.6	< .0001	56.7 ± 95.0	41.5 ± 57.5	30.1 ± 21.8	< .0001
*rs2074356*	GG	GA	AA	*P*-value	GG	GA	AA	*P*-value
Non-drinker	23.2 ± 22.5	23.1 ± 20.4	23.5 ± 21.4	0.0061	21.8 ± 20.7	23.9 ± 25.8	23.4 ± 18.1	0.3471
Drinker	39.8 ± 51.4	30.6 ± 32.6	25.6 ± 15.8	< .0001	56.8 ± 94.7	37.1 ± 45.1	46.5 ± 58.1	< .0001
*rs11066280*	TT	TA	AA	*P*-value	TT	TA	AA	*P*-value
Non-drinker	23.1 ± 22.1	23.3 ± 21.4	23.4 ± 21.1	0.0004	21.9 ± 21.1	23.5 ± 25.2	23.5 ± 17.4	0.2220
Drinker	40.0 ± 52.0	31.7 ± 33.4	26.0 ± 15.0	< .0001	56.8 ± 94.9	41.4 ± 57.5	30.8 ± 22.1	< .0001
*Aspartate aminotransferase (AST, IU/L)*								
*rs77768175*	AA	AG	GG	*P*-value	AA	AG	GG	*P*-value
Non-drinker	23.7 ± 37.8	23.0 ± 10.7	23.0 ± 9.2	0.1942	23.7 ± 16.7	24.3 ± 21.1	23.1 ± 7.9	0.2130
Drinker	24.3 ± 12.6	23.2 ± 13.9	22.4 ± 7.1	< .0001	29.0 ± 20.7	26.4 ± 14.1	22.8 ± 6.6	0.0002
*rs2074356*	GG	GA	AA	*P*-value	GG	GA	AA	*P*-value
Non-drinker	23.7 ± 36.9	22.9 ± 10.6	23.0 ± 9.5	0.1322	23.6 ± 16.2	24.5 ± 22.0	22.8 ± 6.9	0.2021
Drinker	24.3 ± 12.5	23.2 ± 14.4	22.1 ± 7.3	< .0001	29.0 ± 20.6	25.5 ± 12.8	22.5 ± 5.2	< .0001
*rs11066280*	TT	TA	AA	*P*-value	TT	TA	AA	*P*-value
Non-drinker	23.6 ± 38.2	23.0 ± 11.0	23.2 ± 11.0	0.3018	23.7 ± 16.7	24.3 ± 21.1	23.1 ± 7.9	0.2448
Drinker	24.4 ± 12.6	23.3 ± 14.1	22.5 ± 6.7	< .0001	29.0 ± 20.7	26.4 ± 14.1	22.7 ± 6.7	0.0002
*Alanine aminotransferase (ALT, IU/L)*								
*rs77768175*	AA	AG	GG	*P*-value	AA	AG	GG	*P*-value
Non-drinker	21.5 ± 31.0	21.4 ± 15.6	21.7 ± 12.8	0.0502	21.2 ± 20.8	23.0 ± 39.9	21.9 ± 11.6	0.1858
Drinker	23.5 ± 18.0	22.2 ± 20.7	24.5 ± 16.8	< .0001	28.0 ± 24.3	24.6 ± 15.7	21.2 ± 13.1	< .0001
*rs2074356*	GG	GA	AA	*P*-value	GG	GA	AA	*P*-value
Non-drinker	21.6 ± 30.4	21.3 ± 15.5	21.5 ± 12.7	0.0287	21.0 ± 20.3	23.4 ± 41.5	21.6 ± 11.6	0.2294
Drinker	23.5 ± 17.9	22.2 ± 21.3	24.5 ± 16.1	< .0001	28.0 ± 24.2	23.5 ± 14.0	19.6 ± 13.2	< .0001
*rs11066280*	TT	TA	AA	*P*-value	TT	TA	AA	*P*-value
Non-drinker	21.5 ± 31.2	21.4 ± 15.9	21.8 ± 14.8	0.1137	21.2 ± 20.8	23.0 ± 39.9	21.9 ± 11.6	0.2176
Drinker	23.5 ± 17.7	22.3 ± 21.6	23.0 ± 15.2	< .0001	28.0 ± 24.3	24.6 ± 15.7	20.9 ± 13.4	< .0001

Data are expressed as means ± standard deviations. Differences among the genotype groups were assessed by general linear models with adjustment for age, smoking status (non, former, current), BMI, and fasting blood glucose. *HEXA* Health Examinees Study

### Analysis of the association of genetic variants in HECTD4 with type 2 diabetes based on drinking status

These SNPs are expected to be associated with type 2 diabetes; thus, we further explored the effects of these SNPs at *HECTD4* on diabetes. The frequencies of the minor allele homozygotes were very low across the populations. Therefore, the risk effect of rs77768175, rs2074356 and rs11066280 on diabetes prevalence was observed under dominant models to ensure adequate statistical power (Table 5). We showed that the rs77768175 AA genotype, rs2074356 GG genotyp,e and rs11066280 TT genotype in HECTD4 were associated with significantly increased risks of diabetes (by 1.839, 1.746, and 1.723 in the HEXA group and by 1.719, 1.787, and 1.659 in the Ansan–Ansung group, respectively) in drinkers. However, no significant relation of *HECTD4* variants to diabetes risk was observed among nondrinkers in the Ansan–Ansung cohort. Furthermore, we found that the rs77768175 AA genotype, rs2074356 GG genotype, and rs11066280 TT genotype in HECTD4 had increased risks of diabetes by alcohol consumption amounts (low, moderate, and high) in the HEXA group, (Supplementary Table 3). These results indicated that the G-allele of rs77768175, A-allele of rs2074356, and A-allele of rs11066280 weare protective against type 2 diabetes.

**Table 5:** Effects of HECTD4 genotype and alcohol consumption on diabetes

	HEXA		Ansan–Ansung	
*rs77768175*	AA	AG + GG	AA	AG + GG
Non-drinker	1.135 (1.034–1.245)	Ref	1.028 (0.815–1.295)	Ref
Drinker	1.719 (1.487–1.988)	Ref	1.839 (1.302–2.597)	Ref
*rs2074356*	GG	GA + AA	GG	GA + AA
Non-drinker	1.145 (1.042–1.258)	Ref	1.037 (0.820–1.311)	Ref
Drinker	1.787 (1.533–2.084)	Ref	1.746 (1.199–2.544)	Ref
*rs11066280*	TT	TA + AA	TT	TA + AA
Non-drinker	1.107 (1.010–1.214)	Ref	1.027 (0.814–1.294)	Ref
Drinker	1.659 (1.449–1.901)	Ref	1.723 (1.228–2.418)	Ref

Multivariate logistic regression models were adjusted for age, sex, BMI, smoking status (non, former, current). Data are expressed as odds ratios (95% confidence intervals)

### Role of HECTD4 in ethanol-induced liver injury

**Fig. 1:**
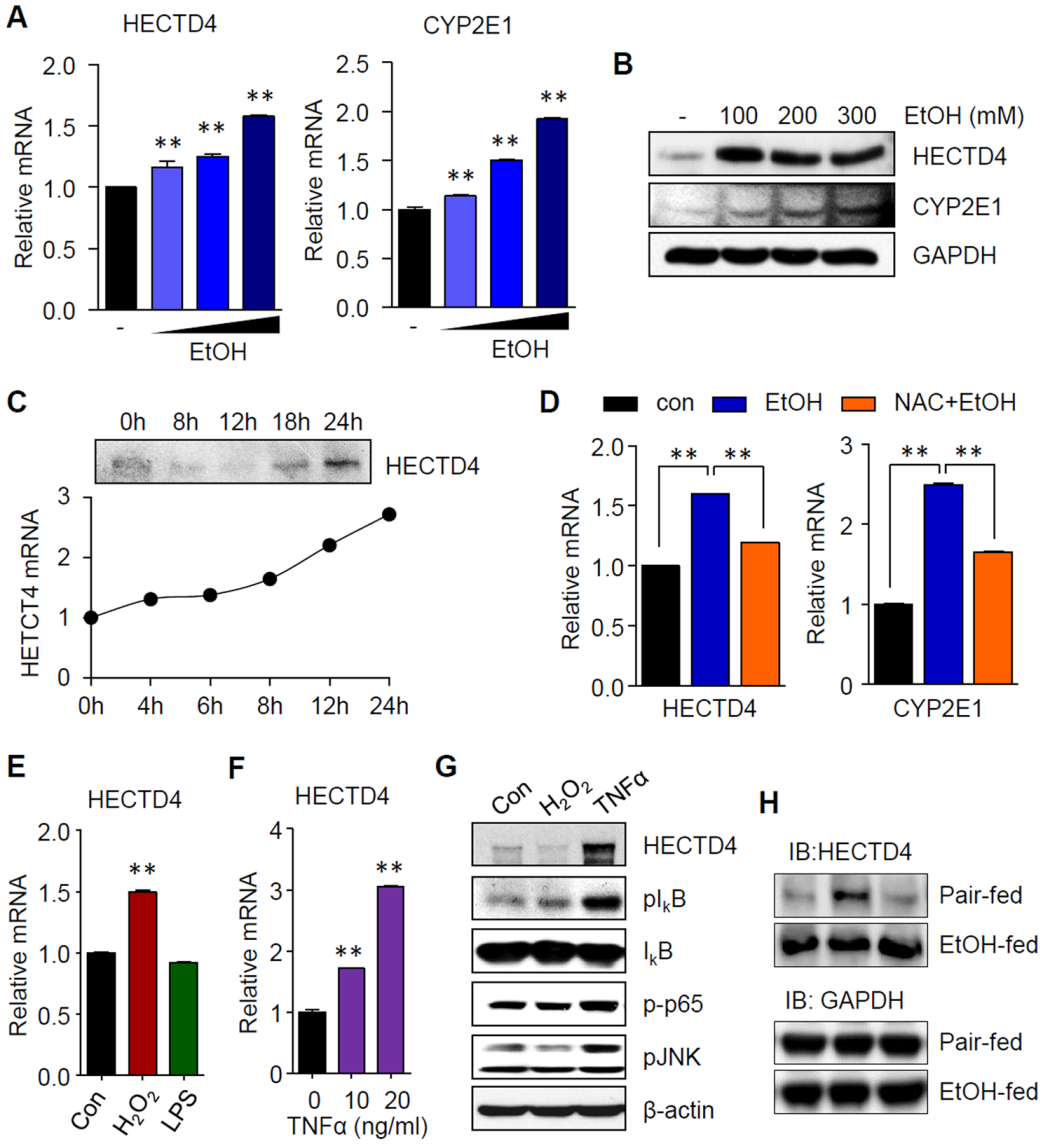
Effect of ethanol treatment on the expression of HECTD4. SK-Hep1 cells were treated with the indicated concentrations of ethanol (100, 200, 300 mM) for 24 h. **A** Relative expression of HECTD4 and CYP2E1 and **B** protein expression following ethanol treatment. **C** mRNA levels of HECTD4 in response to 300 mM ethanol for 0–24 h in cells. **D** SK-Hep1 cells were pretreated with 1 mM NAC for 1 h and then treated with 300 mM ethanol for 24 h, followed by measurement of HECTD4 mRNA by qPCR. **E** and **F** Relative mRNA levels and **G** western blot analysis of HECTD4 levels in cells treated with H_2_O_2_ (200 uM) or TNFα (10 ng/mL) for 24 h. **H** Western blot analysis of HECTD4 protein expression in liver tissue of pair-fed and ethanol-fed mice. The data represent the mean ± SEM. All results are representative of more than three independent experiments. One-way ANOVA with post hoc Tukey’s test, *p < 0.05, **p < 0.01

To investigate the effects of ethanol treatment on HECTD4 expression, we examined the level of HECTD4 in SK-Hep1 cells exposed to ethanol for 24 h. As expected, the induction of CYP2E1, which is responsible for the generation of ROS after ethanol treatment, was significantly elevated in a dose-dependent manner. Similarly, treatment with ethanol resulted in a progressive increase in HECTD4 expression (Fig. 1A and B). In addition, we showed that HECTD4 was highly upregulated at the 24 h time point when SK-Hep1 cells were exposed to ethanol at a concentration of 300 mM over a 24 h period (Fig. 1C). These results indicated that the HECTD4 expression pattern was associated with increased generation of reactive oxygen species (ROS) by ethanol. As expected, *N*-acetylcysteine (NAC) pretreatment reduced the mRNA expression levels of HECTD4 and CYP2E1 (Fig. 1D). Alcohol-induced oxidative stress promotes hepatic inflammation by increasing gut-derived endotoxin lipopolysaccharide (LPS), which increases the production of the pro-inflammatory cytokine tumor necrosis factor α (TNFα) from activated Kupffer cells in liver (Casini 2000). Next, we evaluated the impact of hydrogen peroxide (H_2_O_2_) or LPS on the level of HECTD4 expression using the HepG2 in vitro cellular model of hepatocytes. As shown in Fig. 1E, an increase in HECTD4 expression was observed following treatment with H_2_O_2_ for 24 h. However, there was no change in HECTD4 expression following LPS treatment (Fig. 1E). In response to TNFα stimulation, the phosphorylation levels of JNK, I_k_B, and NF_k_B protein p65 were markedly enhanced compared to those in the untreated control. Similarity, consistent with this, HECTD4 was also significantly upregulated in cells treated with TNFα for 24 h (Fig. 1F and G). Likewise, we observed that HECTD4 expression was strongly elevated in liver from alcohol-fed mice compared with pair-fed mice, indicating a possible involvement of HECTD4 in ethanol-induced liver damage (Fig. 1H). Collectively, these results show that HECTD4 may be closely related to the progression of ethanol-induced hepatosteatosis.

### HECTD4 deficiency alleviates ethanol-induced lipid metabolism

**Fig. 2:**
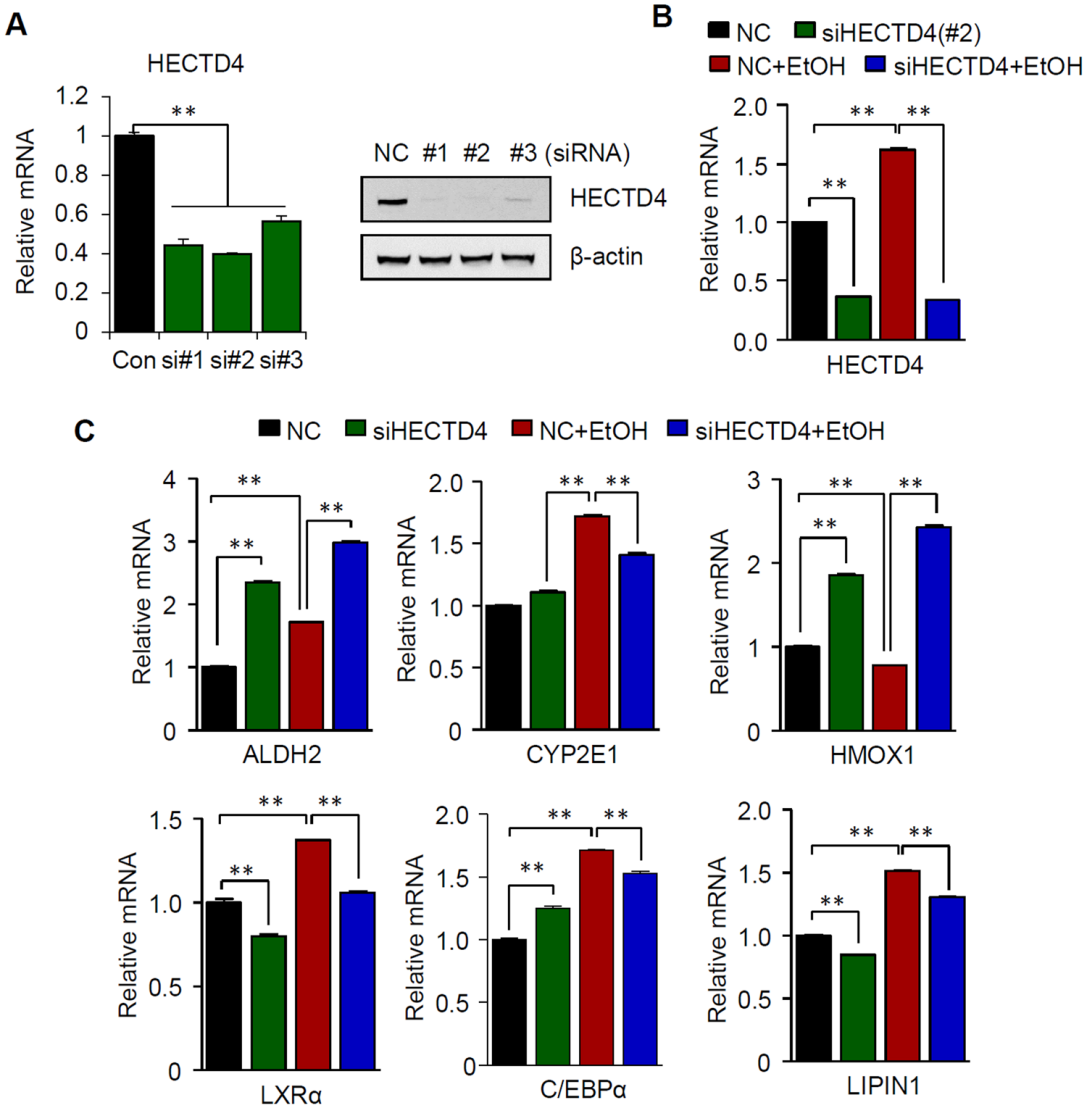
Effect of HECTD4 knockdown. (A and B) HECTD4 mRNA and protein levels were determined in siRNA‐transfected SK-Hep1 cells. (C) SK-Hep1 cells were treated with si-HECTD4 in the presence or absence of ethanol. RT − qPCR analysis of the relative expression of ethanol metabolism- and TG synthesis-related genes. The data represent the mean ± SEM. All results are representative of more than three independent experiments. One-way ANOVA with post hoc Tukey’s test, *p < 0.05, **p < 0.01

To investigate the impacts of HECTD4 on ethanol treatment, we next examined the functional relevance of HECTD4 by performing gene silencing. First, we transfected SK-Hep1 cells with three different HECTD4-specific siRNAs. The qPCR and western blot results confirmed significant downregulation of HECTD4 gene expression compared with control siRNA-transfected cells (Fig. 2A). We next observed the impacts of HECTD4 knockdown on ethanol-induced hepatic steatosis. SK-Hep1 cells were transfected with siRNA-targeting HECTD4 and then incubated in the presence or absence of 300 mM ethanol for 24 h. HECTD4 knockdown blocked increases in HECTD4 expression levels induced by ethanol treatment (Fig. 2B). Furthermore, we found that RNAi-mediated loss of HECTD4 function upregulated the level of ALDH2 expression, whereas CYP2E1 expression was reduced in ethanol-treated cells (Fig. 2C). Acetaldehyde binds to glutathione (GSH) and increases ROS production while reducing GSH (Viña et al. 1980). In addition, the serum levels of heme oxygenase-1 (HMOX1) were lower in patients with acute alcoholic hepatitis compared to healthy controls (Liu et al. 2018). We observed that ethanol treatment produced a significant decrease in HMOX1 mRNA, indicating an impaired antioxidant defense system. However, knockdown of HECTD4 resulted in significantly increased HMOX1 levels, suggesting less oxidative stress induced by ethanol treatment. Notably, the reduction in HECTD4 expression abolished ethanol-enhanced genes involved in hepatic lipogenesis, such as liver X receptor (LXR), CCAAT enhancer-binding protein alpha (C/EBPα), and LIPIN1. These data suggest that alcohol consumption influences the effect of HECTD4 on hepatic triglyceride accumulation through lipogenesis.

## Discussion

In our study, we examined the potential effects of *HECTD4* genetic variants on the prevalence of type 2 diabetes based on alcohol intake. In line with previous studies (Baik et al. 2011; Kim et al. 2016; Yang et al. 2013), we confirmed that three SNPs (rs77768175, rs2074356 and rs11066280) located at an intron of the *HECTD4* gene were significantly associated with alcohol consumption in two independent populations. Thus far, rs671 in *ALDH2* was found to be the most common gene associated with alcohol consumption in East Asian populations. Meanwhile, a GWAS of alcohol consumption in individuals of European ancestry found rs6943555 in *AUTS2* and rs11940694 in *KLB* to be associated with alcohol consumption. (Schumann et al. 2011, 2016). In accordance with these results, we also observed an association with rs671 in *ALDH2,* but genetic polymorphisms of *HECTD4* showed the strongest association with alcohol consumption in our sample.

The association between *HECTD4* variants and type 2 diabetes was previously reported. The rs2074356 SNP had a convincing association with the waist-hip ratio (WHR) (Cho et al. 2009) and HDL-cholesterol (Kim et al. 2011) in Asian populations. Moreover, this SNP was significantly associated with fasting plasma glucose and homeostasis model assessment of β-cell function (Go et al. 2014). In the present study, we found that the three SNPs in the *HECTD4* gene were associated with fasting blood glucose concentrations in the study population and with decreased plasma glucose levels in drinkers. The minor alleles of all three SNPs were obviously associated with decreased multiple metabolic traits, mainly in drinkers, suggesting that *HECTD4* gene variants might influence the risk of type 2 diabetes. In a recent study, minor alleles of the two *HECTD4* variants rs2074356 and rs11066280 were reported to be associated with a reduced thoracic-to-hip circumference ratio (THR) in Korean men (Cha et al. 2015). Consequently, consistent with our results, the results of these studies suggested that the genetic markers of the *HECTD4* gene have an impact on the risk of type 2 diabetes and that these markers showed remarkably consistent associations with multiple metabolic traits.

Several studies have demonstrated that alcohol intake was positively associated with the incidence of impaired insulin secretion (Lee et al. 2017; Tatsumi et al. 2018). Although not yet analyzed in our study, alcohol consumption interacted with genetic risk of type 2 diabetes and was a strong risk factor for blood glucose deterioration (Yu et al. 2019). In a previous study, an interaction between the *ADH1C* genotype and alcohol consumption was observed to influence the risk of type 2 diabetes among women (Beulens et al. 2007). Similarly, Park et al*.* (Park et al. 2018) reported that the *CDKAL1* variants rs7754840 and rs9460546, the *HHEX* variant rs5015480, and the *OAS3* variant rs2072134 interacted with alcohol intake to increase the risk of developing type 2 diabetes due to lower beta-cell function in Koreans. However, Kim et al*.* (Kim et al. 2016) reported that rs2074356 and rs11066280 variants showed no interaction with alcohol consumption regarding the risk of type 2 diabetes in a prospective cohort study. Unlike a previous report, the present study investigated the association between genetic variants in *HECTD4* and type 2 diabetes according to alcohol consumption in two cross-sectional studies. Our results demonstrate that variants in the *HECTD4* gene predispose patients to type 2 diabetes through alcohol consumption. Stratified analyses revealed that the major allele homozygous genotypes of each of the three SNPs are associated with a higher risk of type 2 diabetes than carriers of the minor allele in drinkers, after adjusting for age, BMI, and smoking status. However, we did not find evidence for an association among nondrinkers. Namely, the present findings suggest that the A-allele of rs77768175, the G-allele of rs2074356, and the T allele of rs11066280 may be risk alleles for diabetes, whereas minor alleles of these SNPs represent protective alleles.

Elevated plasma GGT enzymatic activity is positively associated with oxidative stress and the risk of metabolic syndrome (Kunutsor et al. 2015; Lee et al. 2004). We found that individuals with the minor alleles of three SNPs in the *HECTD4* gene had lower GGT levels than those with the major alleles. In view of these results, it is possible that upregulation of HECTD4 significantly contributed to increases in GGT levels as well as TG levels, indicating that *HECTD4* variants are associated with a reduction in HECTD4 gene expression. Our findings provide initial data on the effects of alcohol on HECTD4 expression in a basic cell line study. In this context, future work should focus on characterizing the biological role of the identified genetic variants in the setting of alcohol consumption under physiological and disease conditions.

Chronic alcohol consumption can lead to alcoholic fatty liver, hepatitis, and fibrosis/cirrhosis in the liver. It is well established that ethanol-induced hepatic steatosis, the earliest response to heavy drinking, is caused by increased intrahepatic lipid biosynthesis, enhanced uptake of exogenous fatty acid from the plasma, and decreased fatty acid oxidation and secretion of very low-density lipoprotein (Jeon and Carr 2020; Yang et al. 2019). Accumulating evidence suggests that excessive alcohol intake significantly increases hepatic oxidative stress, causing a reduction in the activity and expression of ALDH2 and leading to hepatic accumulation of acetaldehyde and 4-hydroxynonenal (Hsu et al. 2020; Moon et al. 2006). Moreover, ALDH2 overexpression exerts protective effects against chronic alcohol intake-induced hepatic injuries, including acetaldehyde accumulation, steatosis and inflammation (Guo et al. 2015; Zhong et al. 2015). In concordance with that study, we observed that silencing the HECTD4 gene induced an increase in the mRNA levels of ALDH2, whereas induction of CYP2E1 expression by ethanol was reduced. In addition, HECTD4 knockdown led to decreased levels of LXR and lipin-1, the major activators of hepatic lipogenesis. It was reported that ethanol feeding upregulates hepatic lipin-1, leading to increased lipogenesis, and ethanol-mediated activation of PAP was closely associated with the development of fatty liver in rodents and humans (Hu et al. 2012), supporting the findings of the current study. Therefore, these data suggest that suppression of HECTD4 is likely to improve ethanol-induced hepatic steatosis.

Human HECTD4 (HECT domain E3 ubiquitin protein ligase 4) protein, also known as C12orf51, is found at chromosome 12q24.13. Previous studies have reported that HECT-type E3 ligases are involved in a wide range of human diseases including neurodegenerative diseases, neurological syndromes, and cancers (Bernassola et al. 2008; Wang et al. 2020). Recently, Wan et al*.* (Wan et al. 2020) demonstrated that the expression of HECTD4 was significantly elevated in cholangiocarcinoma tissues and cancerous cells. Nevertheless, little is known about the biological function of HECTD4, and substrates of HECTD4 have not yet been identified. Thus, our data show for the first time, to our knowledge, that HECTD4 levels were elevated in response to ethanol treatment in the liver, which was abolished by antioxidants such as NAC, suggesting that HECTD4 may be positively regulated by oxidative stress and contribute to ethanol-induced liver injury.

## Conclusions

The present study demonstrates that variants in *HECTD4* showed a strong association with alcohol consumption, and the minor allele of these variants conferred protection from, rather than risk for, high consumption. Of note, minor allele carriers of *HECTD4* SNPs (rs77768175, rs2074356 and rs11066280) had significantly lower fasting blood glucose levels than noncarriers in a large Korean population. Furthermore, these variants significantly contributed to reductions in GGT, TG, and WHR in drinkers. Consequently, we found that *HECTD4* polymorphisms had a protective effect regarding diabetes risk among drinkers. Our findings provide additional insights into genetic variations of *HECTD4* associated with alcohol consumption in the pathogenesis of type 2 diabetes.

## Supplementary Information

Below is the link to the electronic supplementary material.

Supplementary file1 (DOCX 51 KB)

## Data Availability

The data used to support the findings of this study are available from the corresponding author upon request.

## Abbreviations

KoGES: Korean Genome and Epidemiology Study
HEXA: Health Examinees Study
SNP: Single-nucleotide polymorphism
BMI: Body mass index
WHR: Waist–hip ratio
TG: Triglyceride
HDL: High-density lipoprotein
AST: Aspartate aminotransferase
ALT: Alanine aminotransferase
GGT: Gamma-glutamyl transferase
ADH1B: Alcohol dehydrogenase 1B
HECTD4: HECT domain E3 ubiquitin protein ligase 4
ALDH2: Aldehyde dehydrogenase2
CYP2E1: Cytochrome P450 2E1
HMOX1: Heme oxygenase-1
