# Supplementary material for: The potential effects of HECTD4 variants on fasting glucose and triglyceride levels in relation to prevalence of type 2 diabetes based on alcohol intake
Source: Arch Toxicol. 2022 Jun 17;96(9):2487–99. doi: 10.1007/s00204-022-03325-y (PMC9325801; doi:10.1007/s00204-022-03325-y)
Supplement: Supplementary file 1 — Supplementary file1 (DOCX 51 KB) [file 204_2022_3325_MOESM1_ESM.docx]

**Supplementary Information**

**The potential effects of HECTD4 variants on fasting glucose and triglycerides levels in relation to prevalence of type 2 diabetes based on alcohol intake.**

Yoo Jeong Lee^1^, Hansongyi Lee^1^, Han Byul Jang^1^, Min-Gyu Yoo^1^, Sumin Im^1^, Soo Kyung Koo^1^*, and Hye-Ja Lee^1^*

^1^Division of Endocrine and Kidney Disease Research, Department of Chronic Disease Convergence Research, National Institute of Health, Cheongju, Chungbuk, 28159, Korea

**Supplementary Figure S1. Flow chart of the study population.**

**Subjects with HECTD4 genotype information were included**

**(n=55,302)**

**Subjects who no information of alcohol consumption were excluded (n=51,289)**

**HEXA (n=58,700)**

**Subjects who diagnosed cancer disease were excluded**

**(n=53,984)**

**Subjects who no information of DM diagnosis were excluded**

**(n=50,028)**

**Final analysis**

**(n=50,028)**

**Subjects with HECTD4 genotype information were included**

**(n=8,840)**

**Subjects who no information of alcohol consumption were excluded**

**(n=8,007)**

**Ansan-Ansung cohort study (n=8,840)**

**Subjects who diagnosed cancer disease were excluded**

**(n=8,834)**

**Subjects who no information of DM diagnosis were excluded**

**(n=7,980)**

**Final analysis**

**(n=7,980)**

| *HEXA* |  |  |  | |  | |  | |  |
| --- | --- | --- | --- | --- | --- | --- | --- | --- | --- |
| CHR | **Position** | **SNP** | | **Gene** | | **Minor** | | **beta** | **P** |
| 12 | 112241766 | rs671 | | ALDH2 | | A | | -10.38 | 5.26E-92 |
| 12 | 112736118 | rs77768175 | | HECTD4 | | G | | -10.23 | 1.45E-91 |
| 12 | 112645401 | rs2074356 | | HECTD4 | | A | | -10.05 | 2.25E-81 |
| 12 | 112817783 | rs11066280 | | HECTD4 | | A | | -8.869 | 4.84E-73 |
| 12 | 111414461 | rs12229654 | | --- | | G | | -7.505 | 8.55E-51 |
| 12 | 111350655 | rs3782889 | | MYL2 | | G | | -5.002 | 1.51E-33 |
| 12 | 111349223 | rs12231049 | | MYL2 | | G | | -4.97 | 3.23E-33 |
| 12 | 111340243 | rs10774610 | | CCDC63 | | C | | -4.777 | 1.04E-31 |
| 12 | 113045654 | rs11066359 | | RPH3A | | T | | -3.718 | 1.11E-28 |
| 12 | 110390979 | rs925368 | | GIT2 | | C | | -7.405 | 1.68E-28 |
| 12 | 111314289 | rs11612727 | | CCDC63 | | C | | -4.622 | 2.81E-28 |
| 12 | 113409176 | rs2072134 | | OAS3 | | A | | -5.768 | 4.48E-28 |
| 12 | 113365621 | rs11066453 | | OAS1 | | G | | -5.099 | 1.92E-25 |
| 9 | 127350074 | rs74737306 | | NR6A1 | | G | | 10.05 | 2.29E-15 |
| 12 | 113921346 | rs79317416 | | --- | | A | | -4.49 | 2.39E-13 |
| 22 | 38891038 | rs18522857 | | DDX17 | | A | | 8.644 | 6.63E-13 |
| 3 | 35337829 | rs117847138 | | LOC101928135 | | A | | 8.89 | 5.74E-12 |
| 3 | 35711942 | rs117621344 | | ARPP21 | | T | | 7.937 | 1.24E-11 |
| 12 | 111993712 | rs7969300 | | ATXN2 | | C | | 1.832 | 1.66E-11 |
| 6 | 55887834 | rs188710923 | | --- | | G | | 9.117 | 3.63E-11 |
| *Ansan-Ansung* | |  | |  | |  | |  |  |
| CHR | **Position** | **SNP** | | **Gene** | | **Minor** | | **beta** | **P** |
| 12 | 112930475 | rs11066325 | | PTPN11 | | C | | -7.956 | 9.58E-83 |
| 12 | 112511913 | rs116873087 | | NAA25 | | C | | -7.844 | 3.88E-81 |
| 12 | 112817783 | rs11066280 | | HECTD4 | | A | | -7.691 | 8.61E-81 |
| 12 | 112736118 | rs77768175 | | HECTD4 | | G | | -7.659 | 1.85E-80 |
| 12 | 112468206 | rs11066132 | | NAA25 | | T | | -7.72 | 4.64E-80 |
| 12 | 112337924 | rs78069066 | | MAPKAPK,  TMEM116 | | A | | -7.645 | 4.58E-79 |
| 12 | 112906873 | rs202040554 | | PTPN11 | | I | | -7.625 | 1.76E-78 |
| 12 | 112645401 | rs2074356 | | HECTD4 | | A | | -7.927 | 5.01E-77 |
| 12 | 112241766 | rs671 | | ALDH2 | | A | | -7.515 | 9.91E-77 |
| 12 | 112230019 | rs4646776 | | ALDH2 | | C | | -7.513 | 1.17E-76 |
| 12 | 112119171 | rs11066001 | | BRAP | | C | | -7.606 | 1.72E-76 |
| 12 | 112574616 | rs12231737 | | TRAFD1 | | T | | -7.397 | 2.56E-76 |
| 12 | 112168009 | rs11066015 | | ACAD10 | | A | | -7.467 | 1.90E-75 |
| 12 | 112627350 | rs144504271 | | HECTD4 | | A | | -7.665 | 2.02E-74 |
| 12 | 112110489 | rs3782886 | | BRAP | | C | | -7.383 | 1.57E-73 |
| 12 | 111836771 | rs1593226460 | | LINC02356 | | I | | -7.384 | 6.36E-71 |
| 12 | 111718231 | rs79105258 | | CUX2 | | A | | -7.308 | 7.88E-68 |
| 12 | 112834586 | rs11537471 | | --- | | G | | -6.651 | 1.81E-66 |
| 12 | 112851282 | rs11066289 | | RPL6 | | T | | -6.423 | 2.93E-63 |
| 12 | 112902324 | rs11066318 | | PTPN11 | | T | | -6.249 | 2.82E-60 |

**Supplementary Table S1. List of top 20 SNPs associated with alcohol consumption (g/day) in each population.**

SNP were tested for the association with alcohol consumption in each GWAS panel using logistic regression analysis after adjustment for age, sex and BMI. Information is based on National Center for Biotechnology Information Database (NCBI) of Single Nucleotide Polymorphisms (dbSNP).

**Supplementary Table S2. Blood liver parameters according to HECTD4 genotype and alcohol consumption.**

| Gamma-glutamyl transferase (GGT, IU/L) | | | | | | | | |  |
| --- | --- | --- | --- | --- | --- | --- | --- | --- | --- |
| *rs77768175* | AA | | AG | | GG | | P-value | |  |
| Non-drinker | | 23.1±22.1 | | 23.2±21.4 | | 23.5±20.8 | | **<.0001** | |
| Low | | 25.1±27.9 | | 25.3±19.1 | | 24.6±14.5 | | **<.0001** | |
| Moderate | | 41.5±44.4 | | 35.0±32.8 | | 28.9±15.4 | | **<.0001** | |
| High | | 71.5±86.1 | | 51.7±73.2 | | 25.0 | | **0.0077** | |
| *rs2074356* | | GG | | GA | | AA | | P-value | |
| Non-drinker | | 23.2±22.5 | | 23.1±20.4 | | 23.5±21.4 | | 0.0061 | |
| Low | | 25.1±27.8 | | 25.3±19.1 | | 24.6±15.6 | | **<.0001** | |
| Moderate | | 41.4±44.2 | | 35.0±33.3 | | 30.2±17.6 | | **<.0001** | |
| High | | 71.3±85.8 | | 52.6±76.5 | | 25. | | **0.0228** | |
| *rs11066280* | | TT | | TA | | AA | | P-value | |
| Non-drinker | | 23.1±22.1 | | 23.3±21.4 | | 23.4±21.1 | | **0.0004** | |
| Low | | 25.1±28.1 | | 25.3±19.5 | | 24.5±14.5 | | **<.0001** | |
| Moderate | | 41.5±44.4 | | 36.1±34.7 | | 28.4±16.3 | | **<.0001** | |
| High | | 71.9±86.9 | | 54.6±68.7 | | 29.6±13.5 | | **0.0028** | |
| Aspartate aminotransferase (AST, IU/L) | | | | | | | | | |
| *rs77768175* | | AA | | AG | | GG | | P-value | |
| Non-drinker | | 23.7±37.8 | | 23.0±10.7 | | 23.0±9.2 | | 0.1942 | |
| Low | | 22.6±10.6 | | 22.4±7.3 | | 22.3±7.0 | | **0.0112** | |
| Moderate | | 24.5±11.4 | | 23.9±19.1 | | 22.9±7.5 | | 0.0684 | |
| High | | 28.0±18.0 | | 25.2±21.7 | | 18.0 | | 0.1069 | |
| *rs2074356* | | GG | | GA | | AA | | P-value | |
| Non-drinker | | 23.7±36.9 | | 22.9±10.6 | | 23.0±9.5 | | 0.1322 | |
| Low | | 22.6±10.5 | | 22.5±7.4 | | 21.8±6.9 | | **0.0182** | |
| Moderate | | 24.5±11.4 | | 24.0±19.9 | | 24.0±9.1 | | 0.1396 | |
| High | | 28.0±18.0 | | 25.5±22.8 | | 18.0 | | 0.2084 | |
| *rs11066280* | | TT | | TA | | AA | | P-value | |
| Non-drinker | | 23.6±38.2 | | 23.0±11.0 | | 23.2±11.0 | | 0.3018 | |
| Low | | 22.7±10.4 | | 22.4±8.5 | | 22.7±7.1 | | **0.0097** | |
| Moderate | | 24.5±11.3 | | 24.0±18.5 | | 22.1±6.4 | | 0.0569 | |
| High | | 28.0±18.2 | | 26.3±19.9 | | 22.6±3.7 | | 0.2943 | |
| Alanine aminotransferase (ALT, IU/L) | | | | | | | | | |
| *rs77768175* | | AA | | AG | | GG | | P-value | |
| Non-drinker | | 21.5±31.0 | | 21.4±15.6 | | 21.7±12.8 | | 0.0502 | |
| Low | | 21.0±17.6 | | 21.6±12.8 | | 25.1±17.2 | | **0.0025** | |
| Moderate | | 24.2±17.4 | | 23.2±29.0 | | 22.9±15.9 | | **0.0036** | |
| High | | 27.7±19.4 | | 22.0±21.6 | | - | | **<.0001** | |
| *rs2074356* | | GG | | GA | | AA | | P-value | |
| Non-drinker | | 21.6±30.4 | | 21.3±15.5 | | 21.5±12.7 | | **0.0287** | |
| Low | | 21.0±17.5 | | 21.6±13.1 | | 24.0±15.1 | | **0.0075** | |
| Moderate | | 24.2±17.3 | | 23.1±30.2 | | 26.6±21.0 | | **0.0094** | |
| High | | 27.6±19.4 | | 22.0±22.6 | | - | | **<.0001** | |
| *rs11066280* | | TT | | TA | | AA | | P-value | |
| Non-drinker | | 21.5±31.2 | | 21.4±15.9 | | 21.8±14.8 | | 0.1137 | |
| Low | | 21.0±17.0 | | 21.5±15.7 | | 24.7±16.6 | | **0.0099** | |
| Moderate | | 24.2±17.2 | | 23.4±28.5 | | 20.8±12.6 | | **0.0131** | |
| High | | 27.7±19.6 | | 23.3±20.3 | | 16.0±5.4 | | **0.0005** | |

Data are expressed as means ± standard deviations. Differences among the genotype groups were assessed by general linear models with adjustment for age, smoking status (non, former, current), BMI and fasting blood glucose.

**Supplementary Table S3. Effects of HECTD4 genotype and alcohol consumption on diabetes.**

|  | HEXA | |
| --- | --- | --- |
| *rs77768175* | AA | AG+GG |
| Non-drinker | 1.135 (1.034-1.245) | Ref |
| Low | **1.681 (1.355-2.085)** | Ref |
| Moderate | **1.684 (1.336-2.123)** | Ref |
| High | **2.381 (1.393-4.067)** | Ref |
| *rs2074356* | GG | GA+AA |
| Non-drinker | **1.145 (1.042-1.258)** | Ref |
| Low | **1.707 (1.366-2.134)** | Ref |
| Moderate | **1.853 (1.441-2.384)** | Ref |
| High | **2.118 (1.237-3.626)** | Ref |
| *rs11066280* | TT | TA+AA |
| Non-drinker | **1.107 (1.010-1.214)** | Ref |
| Low | **1.686 (1.367-2.078)** | Ref |
| Moderate | **1.623 (1.309-2.012)** | Ref |
| High | **1.756 (1.172-2.631)** | Ref |

Multivariate logistic regression models were adjusted for age, sex, BMI, smoking status (non, former, current). Data are expressed as odds ratios (95% confidence intervals).
